# Decoding anomalous thermal transport in magnetic semiconductors

**DOI:** 10.1126/sciadv.adw7332

**Published:** 2026-01-02

**Authors:** Bidesh Biswas, Sourav Rudra, Taishun Manjo, Ashalatha Indiradevi Kamalasanan Pillai, Manisha Bansal, Marian Brännvall, Davide Gambino, Magnus Garbrecht, Tuhin Subhra Maity, Hiroshi Fukui, Björn Alling, Bivas Saha

**Affiliations:** ^1^ Chemistry and Physics of Materials Unit, Jawaharlal Nehru Centre for Advanced Scientific Research, Bangalore 560064, India.; ^2^ International Centre for Materials Science, Jawaharlal Nehru Centre for Advanced Scientific Research, Bangalore 560064, India.; ^3^ Precision Spectroscopy Division, Japan Synchrotron Radiation Research Institute (JASRI), SPring-8, 1-1-1 Koto, Sayo, Hyogo 679-5198, Japan.; ^4^ Sydney Microscopy and Microanalysis, The University of Sydney, Camperdown, NSW 2006, Australia.; ^5^ School of Physics, Indian Institute of Science Education and Research Thiruvananthapuram, Thiruvananthapuram, Kerala 695551, India.; ^6^ Department of Physics, Chemistry, and Biology (IFM), Linköping University, 58183 Linköping, Sweden.; ^7^ Department of Physics, University of Helsinki, P.O. Box 43, FI-00014, Finland.; ^8^ School of Advanced Materials and Sheikh Saqr Laboratory, Jawaharlal Nehru Centre for Advanced Scientific Research, Bangalore 560064, India.

## Abstract

Thermal conductivity in semiconductors typically decreases at high temperatures due to enhanced phonon-phonon interactions. In contrast, certain magnetic semiconductors, such as chromium nitride (CrN), exhibit an increased thermal conductivity above its Néel temperature, predicted to be caused by strong spin-phonon coupling. However, this hypothesis lacks experimental verification and detailed understanding. Here, we present conclusive experimental evidence showing that the enhanced thermal conductivity in CrN arises from anomalous temperature-dependent acoustic phonon lifetimes, driven by a dynamic coupling between spin fluctuations and acoustic phonons in the system. Temperature-dependent inelastic x-ray scattering measurements reveal that acoustic phonon lifetimes in CrN are strongly suppressed near Néel temperature but anomalously increase at higher temperatures. In contrast, the optical phonon lifetime decreases with rising temperature, akin to a nonmagnetic semiconductor, as it remains unaffected by spin-phonon coupling. This finding unveils the microscopic origin of anomalous heat transport behavior in magnetostructurally coupled materials, offering a pathway for effective thermal management in magnetic material–based devices.

## Teaser

Dynamic coupling between spin fluctuations and phonons causes anomalous thermal transport in magnetic semiconductors.

## INTRODUCTION

The mutual interplay among fundamental excitations in materials, such as phonons, magnons, plasmons, etc., constitutes some of the most intriguing phenomena in condensed matter physics (*1*–*4*). One notable outcome of such interplay is the spin-phonon coupling (*5*, *6*). Traditionally, the spin degrees of freedom and phonon excitations are analyzed independently in most materials. However, in magnetic systems, both can be distinctly coupled, especially at high temperatures (*7*). Since the initial demonstration in chalcogenides such as CdCr_2_Se_4_ and CdCr_2_S_4_ (*8*), the spin-phonon coupling has been studied in several material systems such as transition metal oxides, molecular magnets, two-dimensional (2D) layered materials (*9*–*12*), etc. Such spin-phonon coupling is pivotal for generating exotic ground states in magnetic materials and a range of emergent phenomena, such as high-temperature superconductivity, colossal magnetoresistance, magnetostriction, and magnetothermal effects (*13*–*16*). In addition, magnetostructurally coupled materials have attracted much interest for multiferroic and spintronic applications, magnetic resonance imaging, and solid-state qubit-based quantum information processing applications where a long spin coherence time is crucial (*17*–*20*). Therefore, an in-depth understanding of spin-phonon coupling behavior and methods to manipulate spin-phonon coupling is essential.

**Fig. 1.**
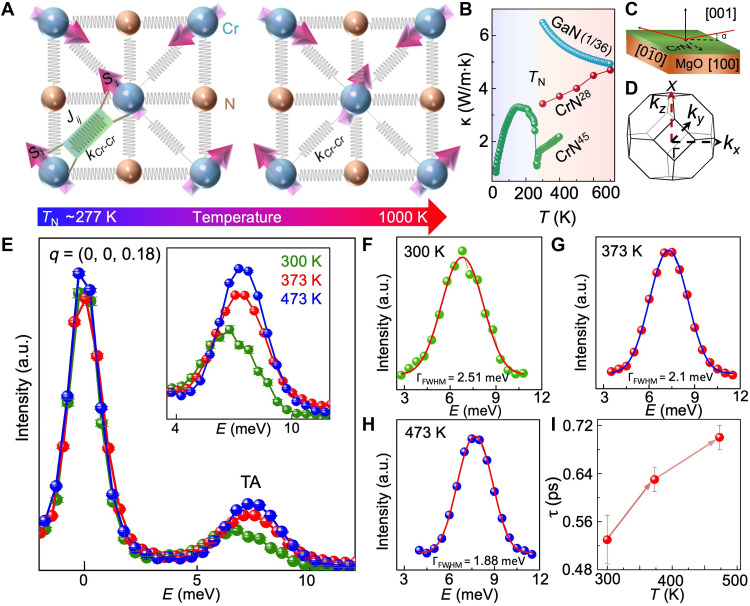
Evolution of dynamic spin-phonon coupling with temperature and its influence on acoustic phonon lifetime. (**A**) Schematic of coupled spin and phonon fluctuations near *T*_N_ in paramagnetic CrN. At higher temperatures (*T > > T*_N_), spin fluctuations and spin-phonon coupling diminish. (**B**) The temperature-dependent thermal conductivity of CrN (*28*, *45*) and nonmagnetic semiconductor GaN (*46*). (**C**) Schematic of IXS measurement setup used for the MgO/CrN film. (**D**) Schematic of measurement direction (Γ-X) in the Brillouin zone of rocksalt CrN. (**E**) Temperature-dependent IXS spectrum at *q* = (0 0 0.18) of the reduced Brillouin zone highlighting the transverse acoustic (TA) mode. The inset shows the broadening of the phonon mode. (**F** to **H**) Voigt function–fitted TA phonon mode of CrN at 300, 373, and 473 K, respectively. (**I**) Temperature-dependent acoustic phonon lifetime increases with rising temperature. a.u., arbitrary units.

Recent theoretical modeling has proposed that chromium nitride (CrN), an archetypical refractory transition metal mononitride, serves as an excellent host for spin-lattice coupling (Fig. 1A) (*21*, *22*). CrN is widely known for its unique magnetic, structural, and electrical phase transition at Néel temperature (*T*_N_) of ~277 K (*23*–*25*). At room temperature, CrN is a paramagnetic semiconductor and turns into an antiferromagnetic metal below *T*_N_. Concomitant to the magnetic and electronic phase transition, the structural symmetry of CrN also changes from rocksalt at high temperature to orthorhombic at low temperature (*T < T*_N_). Theoretical modeling and recent experiments have verified that the phase transition in CrN is initiated by anisotropic magnetic stress, stemming from spin-phonon coupling that distorts the crystal structure and realigns electronic bands (*26*, *27*). Although the influence of spin-phonon coupling on microscopic origin of CrN’s phase transition is well established, the impact of such spin-phonon coupling on the physical properties is less explored.

One of the pronounced manifestations of the spin-phonon coupling in CrN is the anomalous temperature dependence of thermal conductivity above *T*_N_, characterized by a minimum near *T*_N_ and increasing thermal conductivity at higher temperatures, i.e., *T > T*_N_ (*28*–*31*). Such an enhancement in thermal conductivity contradicts the phonon-dominated thermal conductivity of nonmagnetic semiconductors, where thermal conductivity decreases due to enhanced phonon-phonon or Umklapp scattering at high temperatures (*T*), following a *1/T* dependence (*32*, *33*). Prior experiments have demonstrated that magnetic materials with a substantial spin-phonon coupling, such as geometrically frustrated oxides YMnO_3_, LuMnO_3_, ScMnO_3_, and CrN, exhibit such anomalous increase in thermal conductivity with increasing temperature above *T*_N_ that solely emanates from the phonon contributions to the thermal transport (*29*, *34*, *35*). Moreover, for CrN, the anomalous thermal conductivity behavior is observed in bulk powders (*30*) as well as in thin films, highlighting its intrinsic nature. Recent studies on nonmagnetic cubic GeTe have also reported a similar anomalous increase in thermal conductivity, attributed to the hardening of transverse optical (TO) phonon modes and the strengthening of second-nearest-neighbor bonds (*36*). However, the microscopic origin of this anomalous phenomenon in magnetic materials exhibiting strong spin-phonon coupling remains largely unexplored.

Theoretical modeling has predicted that spin-phonon coupling highly influences the acoustic phonon lifetime in paramagnetic CrN (*21*). A combination of atomistic spin dynamics (ASD) (*37*, *38*) and ab initio molecular dynamics (AIMD) simulations modeling results shows that near *T*_N_, the dynamical coupling between spin fluctuations and phonons is substantial in paramagnetic CrN with short-range magnetic ordering, leading to nonadiabatic effects that suppress phonon lifetime. However, the coupling weakens at higher temperatures, enhancing phonon lifetimes. Since the thermal conductivity varies linearly with phonon lifetime, CrN exhibits an anomalous increase in thermal conductivity above *T*_N_. Moreover, as acoustic phonon modes are governed mainly by the Cr─Cr nearest neighbors’ vibrations with Cr atoms having a robust magnetic moment of ~2.8 μ_B_ (*39*), only acoustic phonon is subjected to the spin-phonon coupling. Optical phonon modes, primarily governed by the N-atom vibrations, remain unaffected due to negligible magnetic moment in nitrogen and exhibit a conventional temperature dependence akin to nonmagnetic materials. Hence, CrN is an excellent test bed for investigating spin-phonon coupling and uncovering the underlying causes of its anomalous thermal conductivity.

However, despite the theoretical prediction, the experimental verification of the anomalous phonon lifetime shortening in paramagnetic CrN has not been possible due to the difficulty in synthesizing epitaxial and stoichiometric films and the complexities in measuring temperature-dependent phonon lifetimes (*40*). Nevertheless, recent research has demonstrated that using small chromium flux, optimized substrate temperature, and other growth conditions, phase-pure, epitaxial, and single-crystalline CrN thin film can be deposited on closely lattice-matched substrates that reproducibly exhibit the simultaneous magnetic, structural, and electronic phase transitions (*24*). Besides, recently the inelastic x-ray scattering (IXS) has been used to measure the phonon lifetime of single crystalline ScN (*41*), a sister material of CrN as well. Therefore, with access to high-quality epitaxial CrN films and a phonon lifetime measurement method, we attempt to test the spin-phonon coupling–mediated anomalous phonon lifetime shortening in paramagnetic CrN.

To this end, in this work, we use 2-μm-thick epitaxial single-crystalline CrN films to measure the temperature-dependent phonon lifetimes with IXS. The CrN thin film was deposited inside an ultrahigh-vacuum magnetron sputtering chamber with a base pressure of 1 × 10^−9^ torr and a growth temperature of 700°C on MgO (001) substrate. The CrN film grows as homogeneous and uniform film with (002) orientations on MgO substrates (see section S3 in the Supplementary Materials for detailed high-resolution x-ray diffraction analysis). As the growth conditions of the film used for the IXS measurements are similar to those of the films reported in (*28*), their temperature-dependent thermal conductivities are directly comparable. The IXS measurements are performed at the BL35XU beam line in SPring-8, Japan synchrotron facility. Using an x-ray beam of energy of 21.747 keV, aligned with the Si (11 11 11) reflection, an impressive energy resolution of ~1.5 meV is achieved (*40*). The incident beam angle relative to the CrN film surface was set at ~0.2° to mitigate the signals from the underlying substrate (Fig. 1C). Here, the (311) Bragg plane is referenced for momentum measurements. Since the thermal conductivity of CrN thin film was measured along the [001] direction, the IXS measurements were carried out along the Γ (0 0 0) to X (0 0 1) direction of the rocksalt CrN Brillouin zone (Fig. 1D).

## RESULTS

### Temperature dependence of acoustic phonon lifetime

The temperature-dependent IXS spectra at *q* = (0 0 0.18) of the reduced Brillouin zone (see Fig. 1E) shows a highly intense elastically scattered Rayleigh peak and the transverse acoustic (TA) phonon modes of CrN at ~6 to 8 meV. The phonon modes near the Γ-point are most important for the acoustic phonon lifetime measurements since they predominantly contribute to the thermal conductivity due to their higher group velocities. To determine the phonon lifetime, each TA phonon peak was fitted using the Voigt function [see Fig. 1 (F and H)]. An energy width of 1.5 meV was considered to account for instrumental broadening. In addition, energy broadening due to finite *q*-resolution was included. The total energy broadening, arising from both instrumental energy resolution and finite *q*-resolution, was treated as a convolution of these two effects. This was subsequently eliminated from the experimentally obtained phonon linewidth to obtain the intrinsic phonon linewidth (see the Supplementary Materials for details). The obtained full width at half maximum (FWHM) of phonon linewidth from the fitting is used to calculate the phonon lifetime using the relation τ^−1^ = π.Γ_FWHM_. Our results show that at room temperature (300 K), the TA phonon mode has a lifetime of 0.53 ps. However, as temperature increases, the lifetime of this TA mode increases to 0.63 ps at 373 K and 0.70 ps at 473 K. Such a ~32% increase in the TA acoustic phonon lifetime from 300 K (close to *T*_N_ of ~277 K) to 473 K evidences the dynamic coupling between spin fluctuations and acoustic phonons with a maximum intensity near *T*_N_. As spin-phonon coupling strength reduces at higher temperatures, acoustic phonon scattering decreases, increasing phonon lifetime.

**Fig. 2.**
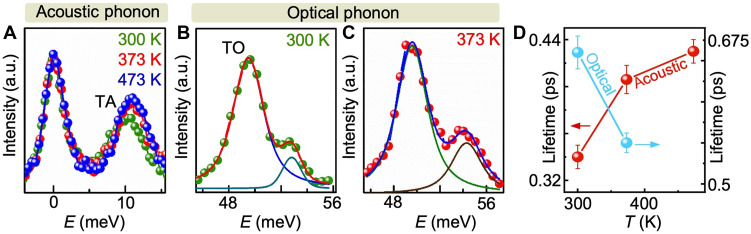
Distinct temperature-dependent behavior of acoustic and optical phonon lifetimes. (**A**) Temperature-dependent IXS spectrum showing the TA phonon mode of CrN at *q* = (0 0 0.26). Similar to *q* = (0 0 0.18), the lifetime of this TA mode also increases with the rise in temperature. (**B** and **C**) The TO phonon mode IXS spectrum at 300 and 373 K, respectively. Small signatures of MgO optical phonon mode are found next to the much more intense CrN TO phonon. (**D**) Temperature evolution of acoustic (red) and optical (cyan) phonon lifetimes. The optical phonon lifetime decreases with rising temperature, while the acoustic phonon lifetime shows an upward trend.

However, since thermal conductivity originates from a cumulative contribution from all phonon modes inside Brillouin zone, acoustic phonon signature of several other *q* points close to zone center is also measured (see Fig. 2A and section S3 in the Supplementary Materials). For the TA mode at *q* = (0 0 0.26), the acoustic phonon lifetime increases from 0.34 ps at 300 K to 0.41 ps at 373 K and further to 0.43 ps at 473 K, following the same trend as *q* = (0 0 0.18) point. Similarly, at *q* = (0 0 0.82), located near the Brillouin zone boundary, the phonon lifetime increases from 0.09 ps at 300 K to 0.17 ps at 473 K (see the Supplementary Materials). A similar trend of increasing phonon lifetime is also observed at other *q*-points (see the Supplementary Materials). Thus, the increase in phonon lifetime above *T*_N_ is observed across all measured TA phonons in CrN, and the enhancement in thermal conductivity at high temperatures is a consequence of this lifetime enhancement.

### Temperature dependence of optical phonon lifetime

A critical approach to further confirming the hypothesis of spin-phonon coupling–mediated thermal conductivity increase is to measure optical phonon lifetimes. The temperature-dependent optical phonon lifetime at *q* = (0 0 0.26) shows that the TO phonon lifetime at 300 K is 0.66 ps, which decreases to 0.55 ps at 373 K. Such a reduction in optical phonon lifetimes is similar to the behavior exhibited by most of the nonmagnetic materials (*41*, *42*) . Since nitrogen atomic vibrations mainly contribute to the TO phonon modes, spin-phonon coupling does not influence the optical phonon modes, which further experimentally verifies the theoretical model.

### ASD-AIMD simulation of phonon lifetime

**Fig. 3.**
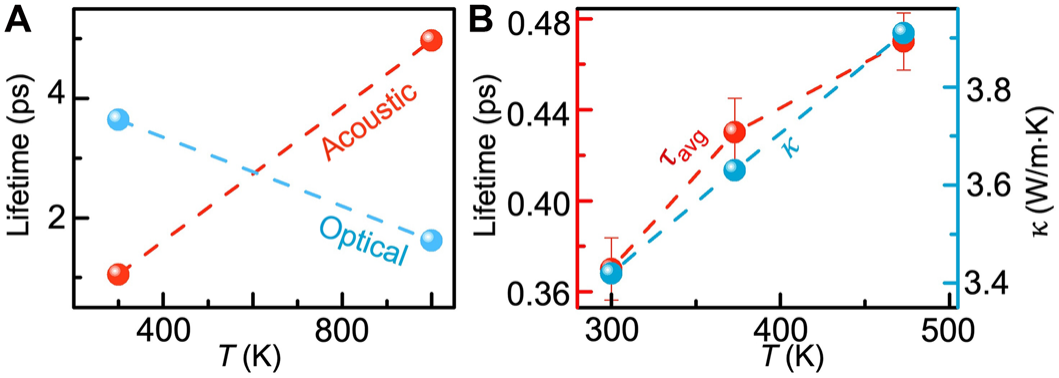
Simulated and experimental phonon lifetimes and its relationship to anomalous thermal conductivity. (**A**) ASD-AIMD simulated temperature-dependent phonon lifetime at *q* = (0 0 0.5) of CrN. The acoustic phonon lifetime anomalously increases with the rise in temperature. Data points are connected by lines to guide the eye and highlight underlying trends. (**B**) Experimentally obtained average acoustic phonon lifetime that increases with temperature. The corresponding thermal conductivity increases accordingly.

Along with the experimental phonon lifetime measurements, ASD combined with AIMD simulations are used further to theoretically emulate the temperature-dependent lifetime of both acoustic and optical phonons. ASD-AIMD is a pioneering approach that considers spin fluctuation and atomic vibration coupling in disordered paramagnetic semiconductors, effectively predicting the temperature evolution of phonon lifetimes. Figure 3A shows the simulated phonon lifetime of paramagnetic CrN at *q* = (0 0 0.5) in the 300 K–to–1000 K temperature range. For acoustic phonon, the lifetime at 300 K is 1 ps, which anomalously increases to 5 ps at 1000 K, resulting in a remarkable 500% enhancement. Conversely, for optical phonons, the room temperature lifetime is 3 ps, which reduces to 1 ps at 1000 K. Therefore, both acoustic and optical phonon lifetimes follow the trend of the experimental results and provide further theoretical understanding.

## DISCUSSION

The origin of anomalous thermal conductivity observed in paramagnetic CrN is elucidated with experimentally measured phonon lifetimes. In the framework of the Boltzmann transport theory under relaxation time approximation, the lattice thermal conductivity of a solid is expressed as (*32*, *43*)

$$k_{L}=\frac{1}{3}\int_{0}^{\omega_{\text{max}}}C_{v}\left(\omega \right)v^{2}\left(\omega \right)\tau \left(\omega \right)\rho \left(\omega \right)\mathrm{d}\omega$$ (1)

where *C_v_*, *v*, τ, and ρ are the specific heat, phonon group velocity, lifetime, and phonon density of states, respectively. In the measured temperature range (300 to 473 K), *C_v_*, *v*, and ρ remain almost unchanged (see the Supplementary Materials for details) (*44*). Therefore, the increased thermal conductivity can only be explained by increase in τ.

For this purpose, the average acoustic phonon lifetime across different *q* points is calculated from the measured lifetime values. At 300 K, the average acoustic phonon lifetime is 0.37 ps (see Fig. 3B). Note that the calculated acoustic phonon lifetimes exceed experimental values since theoretical models do not account additional phonon scattering from defects, ionized impurities, grain boundaries, etc. As the temperature increases to 373 and 473 K, the measured average phonon lifetime increases to 0.43 and 0.47 ps, respectively, representing a ~16 and ~27% increase. Accordingly, the thermal conductivity also increases from 3.4 to 3.8 W/m·K in the same temperature range. Although the temperature-dependent phonon lifetime for the TA mode is experimentally measured here, the change in lifetime also applies to the LA mode due to Cr atom–dominated vibrations. Computational analysis at 300 and 1000 K confirms that, like TA modes, LA modes exhibit increased lifetime with rising temperature (see the Supplementary Materials). Thus, the temperature dependence of acoustic phonon lifetime increase above *T*_N_ explains the origin of anomalous thermal conductivity in CrN.

**Fig. 4.**
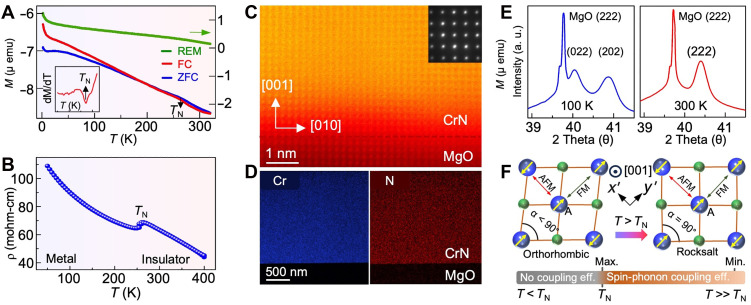
Simultaneous magnetic, structural, and electronic transition in across Néel temperature. (**A**) The *M*-versus-*T* graph showing the paramagnetic-to-antiferromagnetic transition at *T*_N_ of ~277 K. Negative moment in ZFC-FC is due to the diamagnetic MgO substrate’s contribution under the bias field (500 Oe). emu, electromagnetic unit. (**B**) Temperature-dependent electrical resistivity showing low-temperature metal–to–high-temperature insulator transition of CrN at *T*_N_ of ~277 K. (**C**) High-resolution transmission electron microscopy micrograph showing sharp and abrupt film-substrate interface. (**D**) Elemental mapping of Cr and N atoms distributed uniformly throughout the film. (**E**) Temperature-dependent high-resolution x-ray diffraction showing the rocksalt-to-orthorhombic structural transition. (**F**) Schematic showing spin alignment in rocksalt and orthorhombic CrN. The dynamic spin-phonon coupling is maximum in the vicinity of *T*_N_ which gradually vanishes at high temperatures. REM, remanent magnetization.

Last, the concomitant magnetic, structural, and electronic transition in the 2-μm CrN film is investigated to confirm the atomic-scale origin of spin-phonon coupling. Temperature-dependent magnetization (*M-*versus*-T*) measurements show a bifurcation between the zero field–cooled (ZFC) and field-cooled (FC) moments near *T*_N_ of ~277 K, indicating a paramagnetic to antiferromagnetic transition (see Fig. 4A). The dM/dT (inset in Fig. 4A) indicates this magnetic transition more clearly. The temperature-dependent electrical resistivity also shows characteristic low-temperature metal–to–high-temperature insulator transition at ~277 K (Fig. 4B). An increase in resistivity at low temperatures appears because of weak electron localization, as found previously (*24*, *25*). High-resolution transmission electron microscopy analysis reveals a smooth, well-defined interface and confirms high-quality cubic epitaxial growth (Fig. 4C). The elemental mapping in Fig. 4D shows uniform Cr and N distribution, indicating compositional homogeneity. Structural transition, probed by the temperature-dependent high-resolution x-ray diffraction (Fig. 4E), shows that the (222) peak of rocksalt CrN (*Fm*$\bar{3}$*m)* splits into (022) and (202) orthorhombic (*Pmmn*) peaks (*42*) upon the structural distortion around ~277 K, confirming the presence of electric, magnetic, and structural transition in the 2-μm-thick epitaxial single-crystalline CrN film.

The previous theoretical and experimental analyses have confirmed that the simultaneous electric, magnetic, and structural transitions stem from the anisotropic magnetic stress. The antiferromagnetic ordering of CrN is such that two ferromagnetic layers alternate along the [110] direction of rocksalt CrN, as shown in the schematic of Fig. 4F. Consequently, the Cr atom A is antiferromagnetically coupled along the *x*ʹ-direction and ferromagnetically coupled along the *y*ʹ-direction with its nearest neighbor Cr atoms. Since in rocksalt CrN, the Cr─Cr bond length is the same in both directions, it induces magnetic stress, causing the spin state to fluctuate from antiferromagnetic to ferromagnetic and vice versa, facilitated by phonon interactions. As a result, a pronounced *q*-dependent spin-phonon coupling appears near the transition point *T*_N_ in CrN. When the magnetic stress is relieved through structural distortion, it leads to a stable orthorhombic and antiferromagnetic phase at low temperatures (*T < T*_N_), accompanied by a reduction in the dynamic spin-phonon coupling and its correlated impact on phonon lifetime. At higher temperatures also (*T > > T*_N_), the spin-phonon coupling becomes weaker, possibly due to a shorter lifetime of magnetic state or higher amplitude of the atomic displacement. In addition, previous theoretical work has shown that magnetic disorder and spatial magnetic short-range order alone do not reduce the phonon lifetime near *T*_N_ (*21*). A comparison between combined Monte Carlo–AIMD simulations and ASD-AIMD simulations highlighted the critical role of dynamical coupling. A static change in anharmonicity also cannot explain the observed results, as the phonon energy variations with temperature are small (see the Supplementary Materials). Therefore, the spin-phonon coupled acoustic phonon lifetime increase explains the origin of anomalous thermal conductivity in CrN and other strongly magnetostructurally coupled materials.

Our work shows the influence of spin-phonon coupling on acoustic phonon lifetimes, a key factor governing lattice thermal conductivity. Given the critical role of thermal management in ensuring the reliable operation of magnetic storage and processing devices, this work offers valuable insights into the thermal properties of magnetic materials, contributing to the development of targeted strategies for thermal regulation in such systems. Furthermore, the methodology used for calculating acoustic phonon lifetimes serves as a model framework for evaluating finite *q*-resolution–corrected phonon lifetimes in other material systems.

### Summary

In summary, we present conclusive experimental evidence of spin-phonon coupling–mediated anomalous acoustic phonon lifetime shortening near Néel temperature of CrN. Such anomalous acoustic phonon lifetime behavior originates because of dynamic coupling between spin fluctuation and acoustic phonon modes, leading to an unusual rise in thermal conductivity at high temperatures. Compared to acoustic phonon modes, optical phonons remain unaffected by such spin-phonon coupling and exhibit a decrease in lifetime with increasing temperature, a pattern commonly observed in most materials. Our work presents a pioneering report on the microscopic origin of anomalous thermal transport in magnetostructurally coupled materials. It will help explain the unusual temperature-dependent thermal transport observed in magnetically frustrated oxide perovskites and other materials. Moreover, our work highlights the importance of spin-phonon coupling and its influence on the physical properties of magnetic semiconductors, paving the way for innovative device designs with diverse applications.

## MATERIALS AND METHODS

### Sample growth

The 2-μm-thick CrN film was deposited on a 1 cm by 1 cm (001) MgO substrate using an ultrahigh vacuum magnetron sputtering system at 700°C. The deposition chamber has a base pressure of 1 × 10^−9^ torr. The Cr target power was set to 25 W during the deposition. The deposition pressure was maintained at 10 mtorr, and a mixture of Ar and N_2_ gases (99.99999% pure), in a 9:6 ratio, was introduced throughout the deposition. Before deposition, the MgO substrate was ultrasonically cleaned in acetone and methanol solution.

### Estimation of phonon linewidth and phonon lifetime

The phonon linewidth has been estimated by fitting the IXS peak with the Voight function embedded in OriginPro software and Python. A fixed instrumental energy resolution of 1.5 meV has been considered for all the cases. In addition, the energy resolution due to finite *q* effect has been calculated. The total energy broadening, arising from both instrumental energy resolution and finite *q*-resolution, was treated as a convolution of these two effects, assuming that the phonon profile is a rectangular function weighted by *E*^*−*2^.

The profile broadening including finite *q* effect is given by

$$F\left(E\right)=\int_{q_{0}-\frac{q_{\text{window}}}{2}}^{q_{0}+\frac{q_{\text{window}}}{2}}q^{-\beta }f\left(E,\alpha q,1.5\;\text{meV}\right)\mathrm{dq}$$ (2)

where $f\left(E,E_{0},1.5\;\text{meV}\right)$ is the profile function with 1.5-meV FWHM at *E*_0_ (Lorentzian, pseudo-Voigt, etc.), E=αq is the acoustic phonon dispersion (assuming linear relation), and $q^{-\beta }$ is the intensity correction factor (β = 2).

### Computational methods (for dynamic spin-phonon coupling)

The phonon lifetimes are obtained from combined ASD-AIMD simulations as introduced by Stockem *et al.* (*21*). In the ASD step, the orientations of magnetic moments are updated according to the Landau-Lifshitz-Gilbert equation, wherein the effective magnetic field driving the dynamics is dependent on the Hamiltonian. In this work, the Heisenberg Hamiltonian is used, incorporating distance-dependent exchange interactions, thus allowing the atomic positions to influence the evolution of magnetic moment orientations. On the other hand, in the AIMD step, the magnetic moment directions will affect the forces acting on the atoms. Consequently, the ASD-AIMD approach captures the coupling between spin and lattice dynamics. Please refer to the Supplementary Materials for detailed discussion on computational methods.

### Magnetic measurements

The magnetic measurements were carried out in a Quantum Design MPMS3 magnetometer. All samples were demagnetized at room temperature by a proper demagnetized protocol before each measurement. The superconducting magnet of the magnetometer was reset to remove any stray field in the magnetometer before each set of measurements. Magnetization versus temperature was measured for 2 to 320 K. ZFC and FC measurements were done under 500-Oe bias field. The negative value in ZFC-FC moment comes from the diamagnetic contribution of the substrate MgO. The bias field was removed for temperature-dependent remanence measurements which does not have diamagnetic contribution from the substrate.

### Electrical resistivity measurement

Temperature-dependent electrical resistivity of the film was measured using the standard four-probe method in the range of 50 to 400 K. Four equidistant metallic contacts were patterned in a linear geometry on the film surface. The sample was mounted onto a resistivity puck compatible with the Physical Property Measurement System (PPMS). A constant dc current was applied through the outer contacts, while the voltage drop was measured across the inner pair to eliminate contact resistance effects. All measurements were carried out using a commercial Quantum Design PPMS system.

### Synchrotron-radiation x-ray diffraction

Synchrotron radiation temperature-dependent asymmetric ω − 2θ x-ray diffraction measurements are performed to study the structural phase transition. Synchrotron measurements are performed at an x-ray energy of 15 keV, and the scattered signal is detected by a Pilatus 100K 2D detector at the P08 beamline of PETRA-III, Deutsches Elektronen-synchrotron (DESY) in Germany. The Kohzu six-circle diffractometer equipped with Pilatus 100 area detector in P08 beamline is used for the measurements. Anton-Paar DCS 500 cryo-stage was used to regulate the temperature of the samples.

## Supplementary Materials

### This PDF file includes:

Supplementary Materials and Methods Supplementary Text Table S1 Figs. S1 to S12 References
