# Supplementary material for: Decoding anomalous thermal transport in magnetic semiconductors
Source: Sci Adv. 2026 Jan 2;12(1):eadw7332. doi: 10.1126/sciadv.adw7332 (PMC12758556; doi:10.1126/sciadv.adw7332)
Supplement: Supplementary file 1 — Supplementary Materials and Methods Supplementary Text Table S1 Figs. S1 to S12 References [file sciadv.adw7332_sm.pdf]

Science Advances
AAAS

Supplementary Materials for

Decoding anomalous thermal transport in magnetic semiconductors

Bidesh Biswas *et al.*

Corresponding author: Bivas Saha, bsaha@jncasr.ac.in

Sci. Adv. **12**, eadw7332 (2026)
DOI: 10.1126/sciadv.adw7332

This PDF file includes:

Supplementary Materials and Methods
Supplementary Text
Table S1
Figs. S1 to S12
References

1. Materials and Methods

A. IXS Measurement: Inelastic X-ray Scattering (IXS) serves as a powerful technique for investigating energy transfer, specifically the energy variance between the incident and scattered X-ray beams, across arbitrary momentum Q in reciprocal space. The experimental setup closely resembles that of X-ray diffractometers, with a notable exception being the energy scan. In IXS, the determination of q is intricately linked to the scattering angle and the crystal's orientation concerning the incident X-ray beam direction.

Preceding the IXS measurements, a series of diffraction measurements were conducted. The X-ray wavelength (λ) employed was 0.5701 Å, and the incident beam's divergence was approximately 0.02°, attributed to the utilization of a focusing mirror. Importantly, these parameters remained consistent during IXS measurements.

B: Identification and Elimination of signals from MgO substrate: Despite the very low angle of incidence (0.2°), initial IXS measurements showed some contributions from the MgO substrate due to X-ray beam traveling through the substrate side edges. To mitigate the MgO signal during the IXS measurements, Indium (In) metal barrier was placed around the exposed area of the MgO. As a result, the intensity of the MgO-TA and MgO-LA modes diminished, while the intensity of the CrN-TA peaks remained unchanged, allowing for clear identification of the CrN-TA peaks without confusion.

Additionally, the theoretical inelastic spectral function of MgO for each analyser position used during the experiment is simulated, using force constants obtained from the DFT calculations with Quantum ESPRESSO package. The simulated IXS spectra of MgO appears narrower because $\boldsymbol{q}$-resolution and other scattering effects that typically broaden the peak are not considered. These simulated IXS spectra identified the MgO TA and LA phonon modes, enabling us to uniquely distinguish the CrN-TA peaks (Fig. S2).

C. Estimation of phonon linewidth and phonon lifetime at *q*= (0 0 0.18):

At q = (0 0 0.18)/2.73 nm^{-1}, the calculated phonon energy peak E_0 is at 6.93 meV. Assuming a linear phonon dispersion, observable E_{ph} in this q window takes value from 6.95/2.73 × (2.73−0.2) ~ 6.41meV to 6.93/2.73 × (2.73+0.2) ~ 7.43 meV and the width ΔE_{ph} is 1.02 meV. The calculated overall linewidth from the above expression of $F(E)$ is 1.82 meV, which is significantly smaller than the experimental phonon linewidth of 3.10 meV at 300K, at the same q points (Fig. S3).

As the width of 1.82 meV can be approximately expressed as square root of sum of squares ($\sqrt{1.02^2 + 1.5^2}$), the linewidth related to the phonon lifetime was calculated as $\sqrt{3.10^2 - 1.02^2 - 1.5^2}$ = 2.51 meV at 300K (*47*). From this phonon linewidth of 2.51 meV, the phonon-lifetime (τ) has been calculated using the relation $\tau^{-1} = \pi.\Gamma_{\mathrm{FWHM}}$. Similarly, the phonon linewidth related to phonon lifetime has been calculated at other q points at different temperatures. For the measurements at 373 K and 473 K, only the α value was updated, based on the linear E vs. q plots experimentally obtained at the respective temperatures.

D. Computational Methods (For dynamic spin-phonon coupling): The AIMD and ASD steps are conducted as outlined in Stockem et al.(*21*), In the AIMD-step, density functional theory (DFT) calculations are performed using the Vienna ab initio simulation package (VASP) (*48–50*). For the ASD step, atomistic spin-dynamics are implemented using UppASD (*37*). In the ASD simulations, a damping parameter $\alpha = 0.05$ is used and the evolution of magnetic moment directions is calculated with a time step of 0.01 fs. To complete a full ASD step, 100 of these time steps are iterated, resulting in a total duration of 1 fs. The distance-dependent exchange interactions, J_{ij} $(|R_j - R_i|)$, must be parameterized beforehand. Here, the parameterization is based on the results from Lindmaa et al. (*51*) where it was found that the interactions between Cr-Cr pairs can be accurately described using only the first and second neighboring shells. A plot showing the parameterization used in this work can be found in Fig. 1 in the supplementary material of Ref. 21.

The DFT calculations employ projector augmented-wave potentials (*52*, *53*) within the local density approximation (LDA) supplemented with a Hubbard potential (U = 3 eV) to account for strong correlation effects of localized d electrons (*54*). A plane wave energy cutoff of 400 eV is used. The noncollinear magnetic moments are constrained using the method developed by Ma and Dudarev (*55*), with the constraining parameter λ initially set to 5 and later increased to 25, utilizing the resulting wave functions from the first DFT calculation as the starting point for the second iteration. To regulate the temperature, a Nosé thermostat with a time step of 1 fs is employed. The simulations are conducted at 300 and 1000 K with the experimental lattice constants, 4.15245 Å and 4.17361 Å, respectively (*56*). A 2×2×2 cubic supercell (32 Cr and 32 N atoms) and a Monkhorst-Pack (*57*) k-point grid of 2×2×2 *k*-points is used. Phonon lifetimes and frequencies are extracted for the following *q*-points: X/2, X, K', L.

To extract the phonon lifetimes and frequencies, the line widths and peak positions are obtained from a spectral density analysis of the AIMD trajectories (*21*, *58*). The spectral density, $S\,(q, \omega)$, is calculated through a Fourier transform of the scattering amplitude, $A\,(q_\alpha, t)$, where q_α is the wave vector in all symmetrically equivalent directions. The phonon lifetimes, τ, are inversely related to the phonon line widths, Γ. This work uses the full widths at half maximum of the spectral density fitted by a Lorentzian function resulting in the relation $\tau = 1/(2\Gamma)$. The multiplicity in the first Brillouin zone of the phonon's wave vectors is included.

E. Computational Methods (For Phonon Dispersion): Phonon dispersion of rocksalt CrN was calculated using two different approaches utilizing first-principles density functional perturbation theory (DFPT) as implemented in the Vienna Ab initio Simulation Package (VASP) (*50*). In first scenario, to optimize computational efficiency, we employed an 8-atom conventional unit-cell of CrN in the rocksalt phase, consisting of 4 Cr and 4 N atoms. Among the four Cr atoms, two were treated as up-spin and the other two as down-spin. We used the projected-augmented wave (PAW) pseudopotential and the generalized gradient approximation (GGA) for the exchange-correlation functional, with an energy cutoff of 500 eV. Electronic correlation effects were included by applying on-site Coulomb and exchange interactions, specifically Hubbard U and Hubbard J, using the Dudarev formalism. For the phonon calculations, an effective U ($U_{eff} = U - J$) value of 3.06 eV was applied. The force constants were computed with a 2×2×2 supercell, encompassing a total of 64 atoms, and a k-mesh grid of 5×5×5. Phonon mode unfolding and phonon dispersion along the Γ–X direction for the two-atom primitive cell were carried out using the Phonopy software.

In the second case, we have calculated the phonon dispersion of paramagnetic (PM) rocksalt CrN using disordered local spin configurations within a 2×2×2 supercell, comprising 64 atoms

(32 Cr and 32 N). The Cr atoms have randomly distributed up-and-down spins, with no specific magnetic ordering considered. Phonon dispersion was computed using the GGA+U method within the special quasi-random structure (SQS) framework (*59*, *60*), employing density functional perturbation theory (DFPT) as implemented in VASP, including non-analytic corrections at the Γ point. Additionally, the full phonon dispersion along the Γ-X direction was obtained through the unfolding method in Phonopy (*61*). Each phonon branch was averaged across the X, Y, and Z directions, revealing that the dispersions differ slightly along these high-symmetry directions due to variations in spin configurations.

F. TEM Lamella Preparation and Analysis: For TEM sample preparation, a 100 nm Pt protective cap was deposited at the target location with a 5 kV electron beam followed by a 1 µm Pt+C protective cap with a 12 kV Xe beam in Thermo Fisher Scientific Helios Hydra PFIB. Trenching and lift out were done with a 30 kV Xe beam at 60, 15, 4, and 1 nA. Then the sample was welded to a Mo grid using a 30 kV Xe beam Pt weld. Thinning was done with tilt angles of ±1.5° with currents of 300, 100, and 30 pA, checking for electron transparency using a 5 kV electron beam with a secondary electron detector. When the ROI was thin enough it was polished with a 5 kV Xe beam with tilt angles of ±3.5°. Final cleaning was performed with a 2 kV Xe beam with tilt angles of ±5.5°.

G. High-resolution STEM and EDS analysis: STEM images and EDS maps were recorded with an image- and probe-corrected and monochromated Themis-Z 60-300 kV equipped with a high brightness XFEG source and Super-X EDS detector system for ultra-high-count rates, operated at 300 kV. The spatial resolution in STEM mode was 0.7 Å. EDS maps used for atomic % quantification have well above 1 M counts, and k-factor analysis and absorption-corrected background subtraction were employed.

2. Supplementary Text

A. Structural Symmetry of CrN at high temperature: CrN is an interesting material and exhibits structural transition as a function of temperature. In the low-temperature antiferromagnetic and metallic phase ($T<T_N \sim$ 280K) CrN exhibits orthorhombic crystal structure, while at high-temperature ($T> T_N \sim$ 280K) semiconducting and paramagnetic phase, CrN exhibits rocksalt cubic crystal structure. In a recent work (*42*), an interesting polar distortion at the low-temperature antiferromagnetic phase of CrN is also reported. Therefore, structural characterization of CrN across the correlated insulating-metal transition temperature range is extremely important.

In our research on CrN, we have performed a detailed structural characterization of CrN epitaxial films not only with the temperature-dependent synchrotron-radiation x-ray diffraction but also with temperature-dependent Raman spectroscopic studies (as presented in detail in our previous works (*27*). Consistent with the other literature reports (*62*, *63*), our results also show CrN thin films exhibiting an orthorhombic-to-cubic structural transition at the Néel temperature. Moreover, in the present work, our focus has been solely on the high-temperature paramagnetic rocksalt phase of CrN, where dynamic spin-phonon coupling near the Néel temperature causes an anomalous reduction in the acoustic phonon lifetime, and hence, the thermal conductivity. Moreover, our inelastic X-ray scattering measurements performed from 300K to 473K temperature range show no changes in the structural symmetry in CrN in these temperature range (see Fig. S1 for the x-ray diffraction data from 311 Bragg plane performed during the IXS measurements).

Additionally, electronic transport, Seebeck coefficient, thermal conductivity and other measurements from our research as well as in literature (*28*, *64*) also show no sign of any structural change in CrN in our temperature range of interest (300K- 473K).

B. Structural Characterization: The high-resolution X-ray diffractogram (HRXRD) in Fig. S8 shows that the 2 μm-thick CrN film grow with 002 orientation on (001) MgO substrate. From the 2θ peak position, a lattice constant of 4.14 Å is determined for CrN that matches with previous reports. The ω-scan (rocking curve) in the inset yields a very small value of ~ 0.20°, which signifies the nominally single-crystalline nature of the film.

C. Origin of diverse temperature-dependent electrical-resistivity in CrN: The nature of the electronic phase transition in CrN across the Néel temperature has been widely studied and debated. However, a consensus has emerged that the high-temperature cubic (rocksalt) phase of CrN exhibits semiconducting behavior with a narrow bandgap. Several experimental studies have supported this conclusion. For example, scanning tunneling microscopy and resistivity measurements have shown semiconducting characteristics with an energy gap of ~70 meV (*23*), while optical absorption experiments have revealed a direct bandgap of ~0.7 eV (*65*). More recently, resonant photoemission spectroscopy has identified CrN as a charge-transfer insulator in its cubic phase (*66*).

Temperature-dependent electronic transport and optical absorption measurements typically show an activation energy of ~0.6 eV and an optical bandgap of similar magnitude (*28*). Thermoelectric studies have also demonstrated a high Seebeck coefficient in the range of 100 -350μV/K in the high-temperature cubic phase, reinforcing its semiconducting character (*28*, *29*, *45*, *67*).

The earlier controversy surrounding the electronic nature of CrN arose from CrN's extreme sensitivity to stoichiometry. Even slight deviations, such as oxygen contamination or nitrogen vacancies can significantly alter CrN's electronic properties (*68*). These defects can increase conductivity and shift the Fermi level into the conduction band, resulting in metallic-like electrical conductivity behavior at high temperatures. Conversely, in the low-temperature orthorhombic phase, such defects may lead to electron localization and a semiconducting-like temperature dependence of resistivity.

Experimental studies, including those using synchrotron spectroscopy and transport measurements, have shown that the negative temperature coefficient of resistivity observed at low temperatures is primarily due to electronic localization effects (*25*). Depending on the deposition conditions, CrN thin films can exhibit a range of resistivity behaviours in both the high and low temperature phases. In particular, films that show semiconducting behavior (i.e., a negative temperature coefficient of resistivity) in the high-temperature cubic phase are often selected for investigating anomalous thermal transport properties.

D. Phonon-dominated thermal conductivity in CrN: In addition to the phonon contributions, electrons can also contribute to thermal conduction. However, in CrN, the electronic contribution is negligible due to the semiconducting nature of the rocksalt phase and its low carrier concentration ($\sim 2 \times 10^{19}$ cm^{-3}). Our previous study (*28*) quantitatively showed that electrons contribute only about 0.2% to the total thermal conductivity. Therefore, the temperature-dependent thermal conductivity observed in CrN is not driven by electronic effects but is instead governed by anomalous phonon dynamics arising from spin-phonon coupling.

In materials where heat is mainly carried by phonons, thermal conductivity (κ) shows a clear pattern with temperature. At very low temperatures, there are very few phonons and little scattering between them, so κ increases with temperature following a T^3 trend due to the rise in specific heat. As the temperature increases to a moderate range, more phonons are present, but scattering from defects and impurities limits how far they can travel. This causes κ to increase more slowly and eventually reach a peak. At high temperatures, near or above the Debye temperature, phonon-phonon (Umklapp) scattering becomes strong, which reduces heat flow. As a result, κ decreases with temperature, typically following a 1/T behavior.

CrN shows a bell-shaped thermal-conductivity curve in its low-temperature orthorhombic phase, peaking around150K (*29*, *30*, *45*). Above this point κ falls, as expected, because phonon-phonon and defect scattering become stronger. After the material undergoes its coupled magnetic, structural, and electronic transition, the specific heat and phonon density of states change very little, so a further 1/T-style decline in κ should continue, driven by Umklapp processes. Instead, κ starts to rise again, an unexpected trend that points to an unconventional scattering channel, the spin-phonon coupling. Conventional phonon-phonon and defect scattering cannot account for the increase, so we carried out a detailed theoretical study to identify the microscopic mechanism behind this anomalous behavior in CrN.

E. Temperature-dependent Thermal Conductivity of CrN: The thermal conductivity in CrN typically ranges from approximately 1 W/m.K to 6 W/m.K, depending on the stoichiometry and crystalline quality of the sample. Epitaxial and high-quality crystalline CrN with minimal point defects exhibits relatively higher thermal conductivity. In contrast, powdered samples usually exhibit lower thermal conductivity due to enhanced grain boundary and defect scattering. The temperature-dependent thermal conductivity also varies with the crystalline quality of the CrN samples. In epitaxial nominally single-crystalline (as shown in our work), spin-phonon coupling mechanisms dominate over other scattering mechanisms, leading to an increased thermal conductivity with rising temperatures above the T_N. However, in powder samples, spin-phonon coupling is overshadowed by other scattering mechanisms, such as, the grain boundary, dislocation and other types of 2D or 3D defect scattering, resulting in a nearly temperature-independent behaviour of overall thermal conductivity.

F. Changes in C_v: The experimental and theoretical C_v values were obtained from the earlier reported work by Zhou et al (*44*). The theoretical work was calculated using density functional theory with both GGA+U and LDA+U methods. The experimental data was obtained from the NIST-JANAF Thermochemical Tables. According to the literature (*46*), in the measured temperature range, C_p changes from 26 J/mole-atom.K at 300K to 24.5 J/Mole-atom.K at 400 K. Subsequently, the C_p increases slightly to 24.9 J/mole-atom.K at higher temperatures. Assuming C_p and C_v are the same for solid CrN, C_v also changes accordingly. Since the C_p (and C_v) initially decreases from ~26 J/mole-atom·K at 300 K to ~24.5 J/mole-atom·K at 400 K, the increasing thermal conductivity in this temperature range cannot be explained by the decreasing C_p. Further, from 373K to 473K, the thermal conductivity and the TA phonon lifetimes increase by 8% and 9.3%, respectively. But the C_p increases by a tiny amount of ~ 0.8%, which also rules out a C_p-dominated increase in thermal conductivity.

G. Changes in Phonon Group Velocity and Density of States: Currently, there are no existing literature reports on the temperature-dependence of phonon velocities in CrN. However, in general, for crystalline materials, sound velocity (v) (which is intricately related to the acoustic phonon group velocity) depends on the elastic constant (c) and the density of the medium (d)

through the relationship $\upsilon=\sqrt{\frac{C}{d}}$. Since both the elastic constants and density of the material could change as a function of temperature, sound velocity also could vary with changes in temperature. However, most theoretical and experimental evidence shows that sound velocity changes are minimal at low temperatures as phonon populations are low, and the material's properties are dominated by harmonic approximation. Meanwhile, sound velocity decreases significantly in intermediate temperatures as anharmonic phonon interactions increase. Finally, at high temperatures (approaching the Debye temperature), the reduction in the sound velocity typically saturates due to the maximum population of phonons.

For CrN, since we are close to the Debye temperature range (460-480K), we do not expect to see a significant change in the sound velocity. We calculated the TA phonon group velocity near the Γ-point of the Brillouin zone from the experimentally obtained temperature-dependent phonon dispersion. At 300K, the slope of the phonon dispersion yields a group velocity ~ 3930 ± 98 m/s, which increases to ~ 4045 ± 101 m/s at 373 K and ~4090 ± 99 m/s at 473 K. This amounts to a very small change in the TA phonon group velocity in the 300K-473K temperature. Moreover, considering the uncertainties in the linear fitting of the E-vs-q plot, which amount to ±2.5%, the observed increase in group velocity falls within the error margin. Hence, the temperature dependence of the phonon velocity (v) also cannot explain the increase in thermal conductivity at higher temperatures in CrN.

Finally, regarding the phonon density of states (ρ), indeed there are no reports in the literature on the changes in the phonon density of states of CrN as a function of temperature. Our assumption on the minute increase in phonon density of states stems from the fact that the Debye temperature of CrN ranges 460K-480K. Since our measurements were carried out in the 300K-473K temperature range that is close to the Debye temperature, most of the phonon modes are already excited. Therefore, it is highly unlikely that the phonon density of states will change by any significant amount in our case. Additionally, within this regime, the phonon-phonon scattering will be enhanced due to the high density of phonons, which, in turn, will reduce thermal conductivity. Thus, it can't explain the increased thermal conductivity at higher temperature in CrN.

Therefore, our detailed explanations prove that in our measured temperature range, i.e. 300K to 473K, C_v, v, and ρ remain nearly unchanged and cannot account for the increase in the thermal conductivity as a function of temperature. The only parameter that increases significantly, as demonstrated in our IXS measurement, is the phonon lifetimes, which, therefore, causes an increase in the thermal conductivity.

H. Spin-phonon Coupling in acoustic phonon mode: The pronounced spin-phonon coupling observed in acoustic phonons of CrN near the Néel temperature originates from the atomistic nature of the vibrational modes. Acoustic phonons predominantly involve the collective motion of heavier and magnetic Cr atoms, which play a central role in establishing the long-range magnetic order. The vibrational dynamics of these Cr atoms can modulate the exchange interactions between neighboring magnetic moments, thereby giving rise to significant spin-phonon coupling, particularly in the vicinity of the magnetic phase transition.

Conversely, optical phonon modes are primarily characterized by the relative displacement of lighter, non-magnetic nitrogen atoms within the unit cell. Given that N atoms do not contribute to the material's magnetic moment, their vibrational modes do not effectively interact with the spin. As a result, optical phonons do not exhibit spin-phonon coupling. This fundamental

difference in the atomistic origin and magnetic relevance of the phonon modes explains why spin-phonon coupling is restricted to acoustic phonons in CrN.

At temperatures well above the Néel temperature ($T>>T_N$), the strength of spin-phonon coupling again decreases due to two key factors. First, the magnetic state exhibits significantly shorter lifetimes at elevated temperatures, promoting an adiabatic decoupling between the spin and lattice subsystems. Second, the amplitude of atomic displacements increases with temperature, indicating a higher vibrational energy scale. However, this is not matched by a corresponding enhancement in the energy scale of magnetic fluctuations, which was already in a disordered paramagnetic state at 300 K. Together, these effects contribute to the weakening of spin-phonon interactions at high temperatures.

I. Spectral density function: Spectral density function $S(q, \omega)$ of CrN obtained through ASD-AIMD simulations are presented in Fig. S5 and Fig. S6. The spectral density analysis reveals slight temperature-dependent shifts in phonon energies for both transverse acoustic and optical modes. Specifically, a minor increase in acoustic phonon energy and a decrease in optical phonon energy are observed with rising temperature. These shifts are relatively small and are primarily attributed to temperature-induced changes in lattice parameters.

J. Phonon lifetime of Longitudinal Phonons: The temperature-dependent lifetimes of longitudinal phonons were extracted at q = (0, 0, 0.5) at 300 K and 1000 K using ASD-AIMD simulation (Table. 1). As anticipated, the phonon lifetime of LA phonons exhibits a similar trend to that of TA phonons, indicating an increase in lifetime with decreasing temperature. In contrast, the LO phonon lifetime remains unaffected by dynamic spin-phonon coupling and decreases with increase in temperature.

Temperature	Phonon lifetime (ps)	
	LA	LO
300 K	3.5587	4.4053
1000 K	4.9261	4.2553

Table S1: **Simulation of Temperature Effects on LA and LO Phonon Lifetimes**. Simulated temperature-dependent phonon lifetime for longitudinal phonon modes in CrN at q = (0, 0, 0.5). Similar to the transverse acoustic phonon, the lifetime of longitudinal acoustic (LA) phonon increased with the increase in temperature, due to the dynamic spin-lattice coupling. For the longitudinal optical (LO) phonon, phonon lifetime decreases with the increase in temperature like most materials not having a string spin-phonon coupling.

K. Phonon Lifetimes at Different *q*-points:

K.1 Temperature-dependent transverse acoustic phonon lifetimes at q = (0 0 0.26). The phonon lifetime at 300K is 0.34 ps which anomalously increases to 0.43 ps at 473 K (see Fig. S9).

K.2 Temperature-dependent transverse acoustic phonon lifetimes at q = (0 0 0.21). Here the phonon lifetime at 300 K is 0.31 ps which anomalously increased to 0.38 ps at 473 K (see Fig. S10).

K.3 Temperature-dependent transverse acoustic phonon lifetimes at q = (0 0 0.82). Here the phonon lifetime at 300 K is 0.09 ps which anomalously increases to 0.17 ps at 473 K (see Fig. S11).

M. Difference in theoretical and experimental phonon lifetimes: The difference between the theoretical and experimental thermal conductivity values in CrN appears as the theoretical simulations performed in our work considered only the spin-phonon coupling as the main scattering mechanism in calculating the phonon lifetime (*21*). In contrast, the real experimental system involves additional scattering mechanisms, such as electron-phonon interactions, defect/impurity-phonon scattering, grain boundary-phonon scattering etc., which were not included in the theoretical calculations.

While spin-phonon coupling tends to enhance phonon lifetime with increasing temperature, the other scattering mechanisms, such as electron-phonon, defect/impurity, and grain boundary interactions contribute to a reduction in phonon lifetime as temperature rises. Despite these competing effects, the dominance of spin-phonon coupling in the system leads to an overall increase in phonon lifetime, consistent with experimental observations. Therefore, the magnitude of the phonon lifetime increase is lower in experiments compared to the theoretically predicted values due to the omission of other scattering processes in the simulation.

N. Limited *q*-point sampling: Inelastic x-ray scattering (IXS) measurements were performed along the Γ (0 0 0) to X (0 0 1) direction of the rocksalt CrN Brillouin zone by referencing the (311) Bragg plane, with data collected using 12 fixed-position analyzers. Due to the fixed geometry, not all q-points align with the high-symmetry direction. Moreover, for thermal conductivity analysis, phonons near the Γ-point of the Brillouin zone are particularly relevant due to their high group velocity. Therefore, the analysis focused on q-points near the zone centre, which adequately explains the observed anomalous thermal conductivity behavior.

O. Optical phonon lifetime at 473K: The optical phonon was not included in the analysis at 473 K due to experimental limitations. Specifically, the Indium (In) metal used to suppress the MgO substrate signal from the substrate-sides started to melt at this higher temperature, which compromised the spectral quality. Although optical phonons could still be detected, accurately fitting the spectrum and extracting reliable phonon lifetimes became difficult.

P. Grain boundary and defects scattering contribution to thermal conductivity: Along with spin-phonon coupling, the average phonon lifetime can have contribution from grain boundary scattering, defect scattering and phonon-phonon Umklapp scattering.

These mechanisms collectively determine the total phonon scattering rate through Matthiessen's rule:

$$\frac{1}{\tau_{\mathrm{tot}}} = \frac{1}{\tau_{\mathrm{GB}}} + \frac{1}{\tau_{\mathrm{def}}} + \frac{1}{\tau_{\mathrm{U}}} + \frac{1}{\tau_{\mathrm{sp}}} \qquad \text{(S1)}$$

For CrN at 300 K, the average acoustic phonon lifetime is approximately $\tau \approx 0.37$. Assuming τ directly corresponds to the scattering time, the phonon mean free path (MFP) at room temperature can be estimated as:

$$\text{MFP} = v \cdot \tau = 3930 \text{ m/s} \times 0.37 \text{ ps} = 0.75 \text{ nm}$$

Where v is the group velocity at room temperature. This MFP is significantly smaller than the average grain size in CrN (~30–40 nm). Assuming the average grain size of 30 nm, the phonon scattering time due to grain boundary scattering is:

$$\tau_{GB} = L/v = \frac{30\ nm}{3930\ m/s} \approx 7.63 \text{ ps}$$

Since $\tau_{GB} \gg \tau$, grain boundary scattering contributes negligibly to the total scattering at room temperature. This is expected, as phonon transport becomes insensitive to grain size when the MFP is much shorter than the grain size, making grain boundary scattering relevant only at lower temperatures.

Defect-induced scattering can be described by the Rayleigh-type relation:

$$\frac{1}{\tau_{def}} = A\omega^4 \qquad \text{(S2)}$$

where A characterizes the level of disorder. For a moderately disordered system with $A = 10^{-42}$ s^3 and phonon frequency ω = 2.5 THz, the corresponding lifetime is:

$$\tau_{def} \approx 25.6 \text{ ns}$$

which are orders of magnitude larger than the observed average phonon lifetime. Even with increased disorder, τ_{def} remains substantially longer, indicating that defect scattering is also negligible.

To evaluate the effect of grain-boundary and defect scattering on thermal transport, the temperature-dependent thermal conductivity was calculated with and without considering these scattering mechanisms. At 300 K, the total thermal conductivity is 2.97 W m^{-1} K^{-1}, increasing slightly to 3.10 W m^{-1} K^{-1} when grain-boundary and defect scattering are excluded. Similarly, at 373 K, the value rises from 3.45 W m^{-1} K^{-1} to 3.65 W m^{-1} K^{-1} upon neglecting these effects (Fig. S12). The combined contribution of grain-boundary and defect scattering thus accounts for only about 5-6% of the total thermal conductivity. The observed increase with temperature underscores the dominant role of spin–phonon coupling in governing the transport behavior, while the calculated values show good agreement with experimental reports for films grown under comparable conditions (*28*). Although the thermal conductivity was estimated using the measured TA phonon lifetime, group velocity, and specific heat, a comprehensive evaluation would require phonon properties across the entire Brillouin zone. Hence, while the model successfully captures the experimental trend of increasing thermal conductivity with temperature, direct quantitative comparisons between the calculated and experimental values should be made with caution.

Thus, phonon transport in CrN within the 300-473 K range is predominantly governed by Umklapp scattering and spin-phonon interactions. The negligible influence of grain boundary and defect scattering further supports that the anomalous thermal conductivity observed in CrN is primarily driven by strong spin-phonon coupling, which counteracts the expected reduction from Umklapp scattering.

3. Supplementary Figures

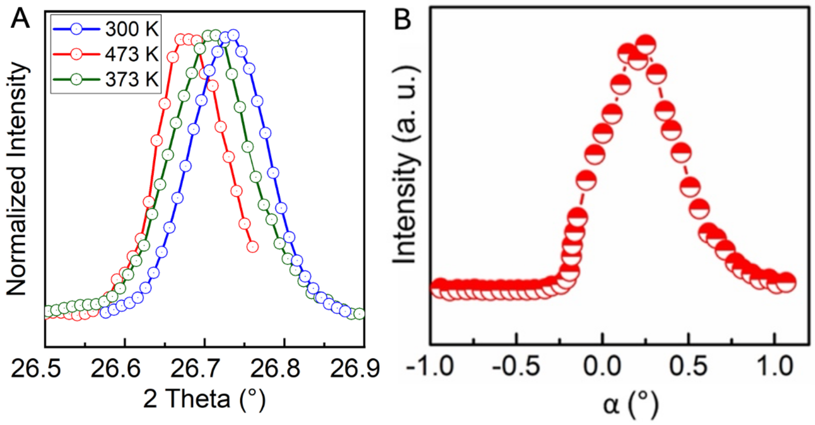

Fig. S1: High-Resolution X-Ray Diffraction (HRXRD) analysis of the 311 Bragg peak. (**A**) HRXRD measurements were carried out on the (311) reference plane of rocksalt CrN at 300 K, 373 K, and 473 K. The diffraction peak remains well-defined across all temperatures, exhibiting a gradual leftward shift with increasing temperature. This shift indicates a thermal expansion of the lattice. (**B**) Peak intensity variation with Angle of incidence (α). The intensity is maximum around α ~0.2°.

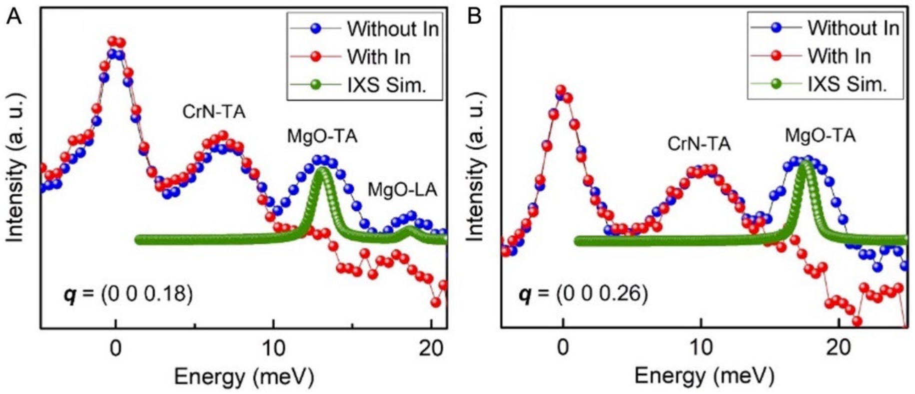

Fig. S2: **Suppression of MgO signal using Indium metal.** (**A**) IXS-spectra at q = (0 0 0.18) with and without the In-metal cover around the MgO substrate edges. Along with the narrow angle-of-incidence, the In-metal cover eliminates the unwanted signal coming from the MgO substrate edges. The simulated IXS of MgO is further utilized to identify the signal contribution from the substrate. (**B**) IXS-spectra at q = (0 0 0.26) with and without the In-metal cover. Here, also CrN-TA phonon peak can be identified uniquely.

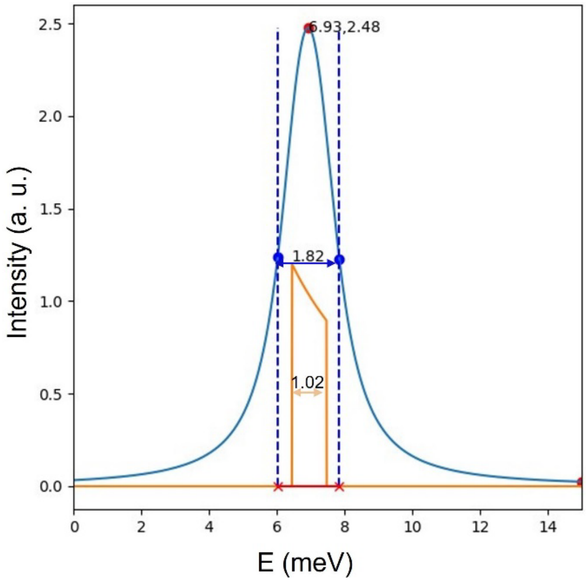

Fig. S3: Finite *q*-resolution correction. The E^{-2}-weight rectangle phonon shape observed in the q window (orange) and that convoluted by 1.5-meV Lorentzian (teal).

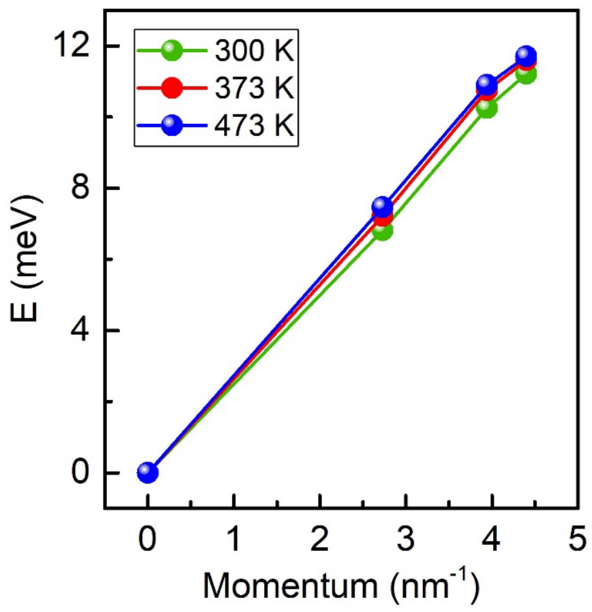

Fig. S4: Temperature-dependent phonon dispersion. Temperature dependent phonon dispersion plot, which yields the phonon group velocity of CrN. The phonon energies changes by a small amount, approximately 0.61 meV, in the temperature range of 300 K to 473 K, effectively ruling out the influence of anharmonicity on the temperature-dependent thermal conductivity of CrN.

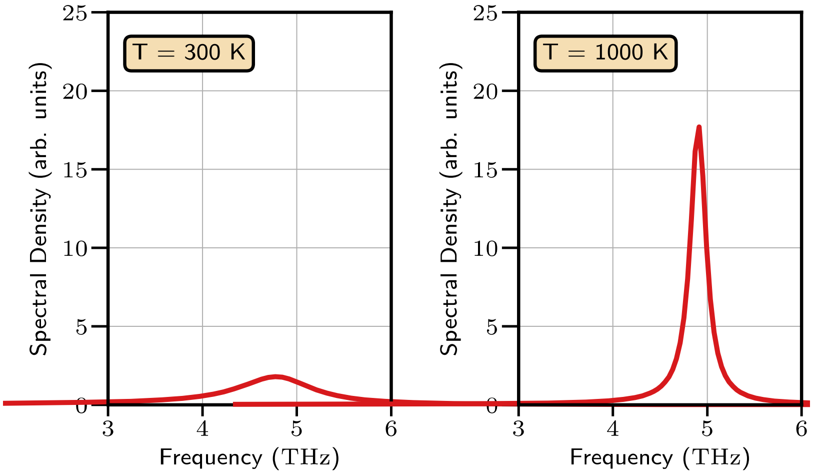

Fig. S5: **Spectral density function of Transverse Acoustic (TA) phonon.** Spectral density function of transverse acoustic mode for $q = (0\ 0\ 0.5)$ at 300 K and 1000 K, respectively, are presented. The phonon energies are obtained from the peak fitting of the spectral density function with Lorentzian function. There is an energy increase of approximately 0.6 meV from 19.7 meV (4.77 THz) at 300 K to 20.3 meV (4.91 THz) at 1000 K.

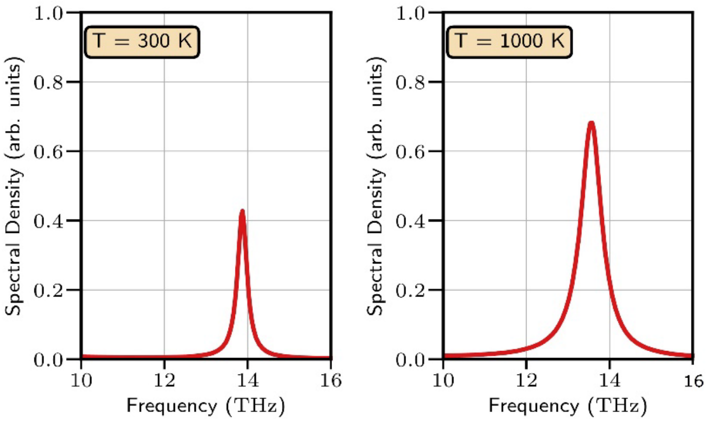

Fig. S6: Spectral density function of Transverse Optical (TO) phonon. Spectral density function of the transverse optical mode for q = (0 0 0.5) at 300 K and 1000 K, respectively, are shown. Phonon energies are obtained from the peak fitting of the spectral density function with Lorentzian function. The optical mode phonon energy decreases from 57.4 meV (13.88 THz) at 300 K to 56.2 meV (13.58 THz) at 1000 K, which is a decrease of approximately 1.2 meV. The observed shifts in phonon frequencies with temperature for both acoustic and optical modes are small and can be attributed to the different lattice parameters employed at different temperatures.

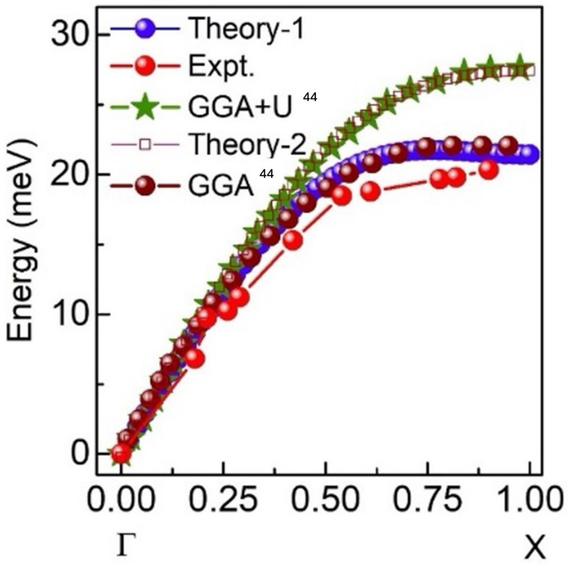

Fig. S7: **Comparison of theoretically simulated and experimentally obtained phonon dispersion.** Phonon dispersion of CrN along the (Γ-X) direction, comparing theoretical simulations and experimental measurements. The blue spheres represent theoretically simulated phonon dispersion using VASP consider an 8-atom unit cell (Theory-1). The hollow red square represents the phonon dispersion simulated through SQS method (Theory-2). The green stars and maroon circles correspond to the phonon dispersions simulated by Zhou et al., (*44*) using the GGA+U and GGA methods, respectively (*reprinted with permission from American Physical Society, https://doi.org/10.1103/PhysRevB.90.184102*). The experimental data are depicted by red spheres.

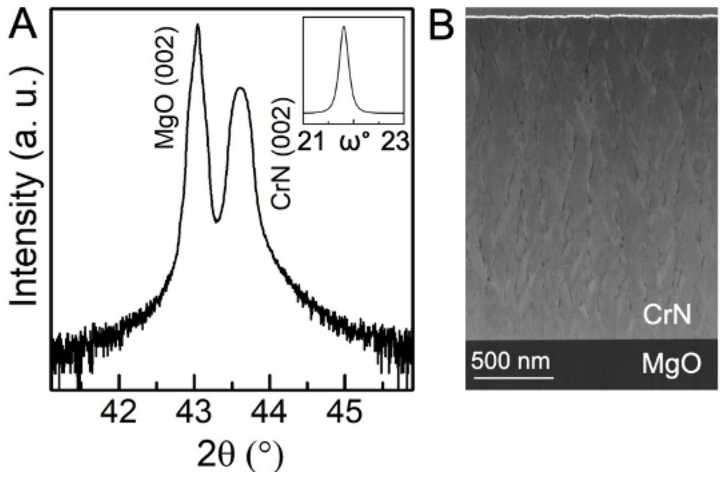

Fig. S8: Structural analysis of CrN with HRXRD, and Transmission Electron Microscopy (TEM). (**A**) High-resolution X-ray diffractogram shows 002 oriented CrN on MgO (002) substrate. The inset shows ω-scan with a very small value of Δω ~ 0.20°. (**B**) High-angle annular dark-field scanning transmission electron microscopy image (HAADF-STEM) shows 2 μm thick CrN film containing nanometer sized voids along extended defects.

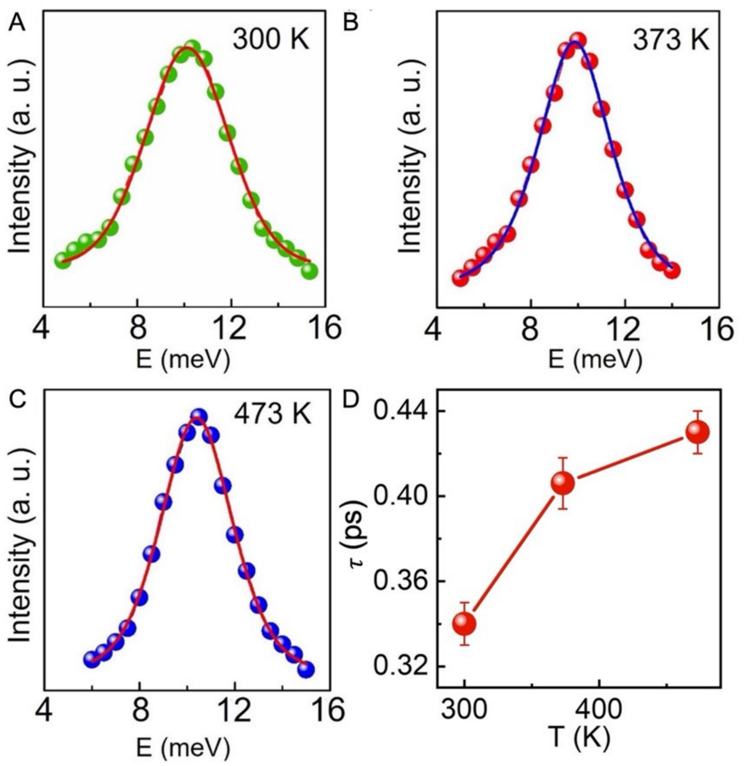

Fig. S9: Temperature-dependent phonon lineshape and lifetime at q = (0 0 0.26). Experimental (circle) and fitted (solid line) acoustic phonon linewidth at q = (0 0 0.26) at 300 K (**A**), 373 K (**B**), and 473 K (**C**). (**D**) Calculated phonon lifetimes at different temperatures. The phonon lifetime increases with the increase in temperature.

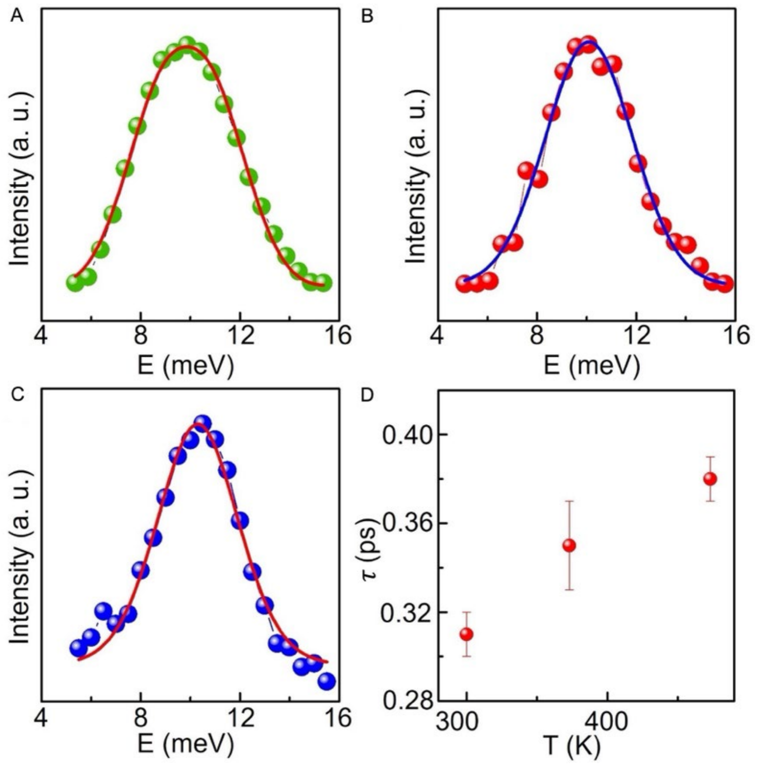

Fig. S10: Temperature-dependent phonon lineshape and lifetime at q = (0 0 0.21). (**A**) Temperature-dependent IXS spectrum at q = (0 0 0.21) of the reduced Brillouin zone highlighting the transverse acoustic mode. Voigt function-fitted TA phonon mode of CrN at 300 K (**A**), 373 K (**B**), and 473 K (**C**), respectively. (**D**) Calculated temperature-dependent phonon lifetimes that increase with the rise in temperature.

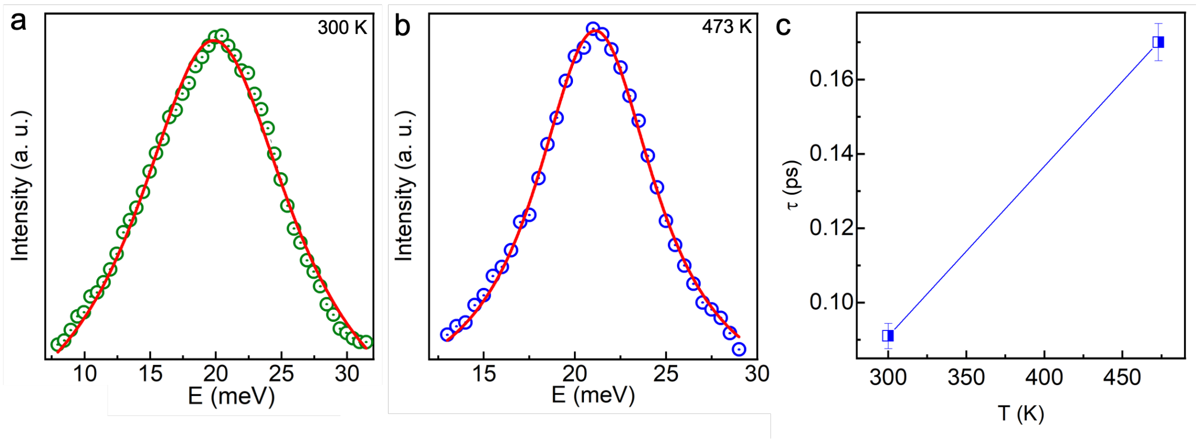

Fig. S11: Temperature-dependent phonon lineshape and lifetime at *q* = (0 0 0.82). Voigt function-fitted TA phonon mode of CrN at *q* = (0 0 0.82) measured at 300 K (**a**) and 473 K (**b**). Like the other *q* points, phonon lifetime here also increases from 0.09 ps at 300 K to 0.17 ps at 473 K (**c**). Unfortunately, due to time constraints during the IXS measurement, *q* = (0 0 0.82) measurement at 373K could not be performed.

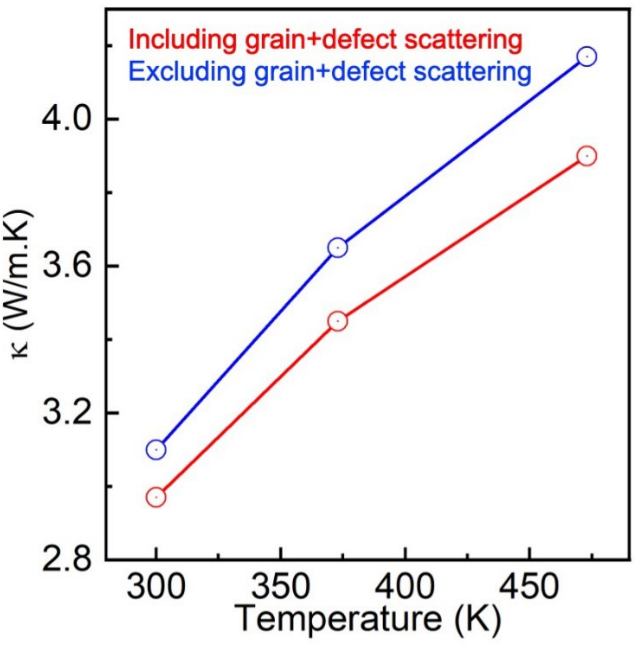

Fig. S12: Grain-boundary and defect scattering effect on thermal conductivity. Theoretically modelled total thermal conductivity (red line) compared with that obtained without considering the grain boundary and defect scattering effects. The total thermal conductivity increases by approximately 5-6% when the grain boundary and defect scattering effects are excluded, indicating their minimal contribution.

REFERENCES

1. Y. J. Bae, J. Wang, A. Scheie, J. Xu, D. G. Chica, G. M. Diederich, J. Cenker, M. E. Ziebel, Y. Bai, H. Ren, C. R. Dean, M. Delor, X. Xu, X. Roy, A. D. Kent, X. Zhu, Exciton-coupled coherent magnons in a 2D semiconductor. *Nature* **609**, 282–286 (2022).

2. D. Q. To, Z. Wang, Y. Liu, W. Wu, M. B. Jungfleisch, J. Q. Xiao, J. M. O. Zide, S. Law, M. F. Doty, Surface plasmon-phonon-magnon polariton in a topological insulator-antiferromagnetic bilayer structure. *Phys. Rev. Mater.* **6**, 085201 (2022).

3. D. I. Khomskii, G. A. Sawatzky, Interplay between spin, charge and orbital degrees of freedom in magnetic oxides. *Solid State Commun.* **102**, 87–99 (1997).

4. D. H. Moseley, S. E. Stavretis, K. Thirunavukkuarasu, M. Ozerov, Y. Cheng, L. L. Daemen, J. Ludwig, Z. Lu, D. Smirnov, C. M. Brown, A. Pandey, A. J. Ramirez-Cuesta, A. C. Lamb, M. Atanasov, E. Bill, F. Neese, Z.-L. Xue, Spin–phonon couplings in transition metal complexes with slow magnetic relaxation. *Nat. Commun.* **9**, 2572 (2018).

5. R. D. Mattuck, M. W. P. Strandberg, Spin-phonon interaction in paramagnetic crystals. *Phys. Rev.* **119**, 1204–1217 (1960).

6. M. W. Wu, J. H. Jiang, M. Q. Weng, Spin dynamics in semiconductors. *Phys. Rep.* **493**, 61–236 (2010).

7. J. Fransson, D. Thonig, P. F. Bessarab, S. Bhattacharjee, J. Hellsvik, L. Nordström, Microscopic theory for coupled atomistic magnetization and lattice dynamics. *Phys. Rev. Mater.* **1**, 074404 (2017).

8. E. F. Steigmeier, G. Harbeke, Phonons and magnetic order in ferromagnetic $CdCr_2Se_4$ and $CdCr_2S_4$. *Phys. kondens. Materie* **12**, 1–15 (1970).

9. A. Tiwari, D. C. Kakarla, B. Poojitha, P. Sahoo, H. L. Liu, A. Dixit, C. W. Wang, T. W. Yen, M.-J. Hsieh, J.-Y. Lin, J. Krishnamurthy, Y. C. Lai, H. Chou, T. W. Kuo, A. Pal, H. D. Yang, Spin-phonon-charge coupling in the two-dimensional honeycomb lattice compound $Ni_2Te_3O_8$. *Phys. Rev. B* **108**, 075113 (2023).

10. C. Cazorla, O. Diéguez, J. Íñiguez, Multiple structural transitions driven by spin-phonon couplings in a perovskite oxide. *Sci. Adv.* **3**, e1700288 (2017).

11. L. Gu, R. Wu, Origins of slow magnetic relaxation in single-molecule magnets. *Phys. Rev. Lett.* **125**, 117203 (2020).

12. M. Ganzhorn, S. Klyatskaya, M. Ruben, W. Wernsdorfer, Strong spin–Phonon coupling between a single-molecule magnet and a carbon nanotube nanoelectromechanical system. *Nat. Nanotechnol.* **8**, 165–169 (2013).

13. B. Keimer, S. A. Kivelson, M. R. Norman, S. Uchida, J. Zaanen, From quantum matter to high-temperature superconductivity in copper oxides. *Nature* **518**, 179–186 (2015).

14. T. Ideue, T. Kurumaji, S. Ishiwata, Y. Tokura, Giant thermal Hall effect in multiferroics. *Nat. Mater.* **16**, 797–802 (2017).

15. M. C. Weber, M. Guennou, D. M. Evans, C. Toulouse, A. Simonov, Y. Kholina, X. Ma, W. Ren, S. Cao, M. A. Carpenter, B. Dkhil, M. Fiebig, J. Kreisel, Emerging spin–phonon coupling through cross-talk of two magnetic sublattices. *Nat. Commun.* **13**, 443 (2022).

16. A. P. Roy, J. Ss, V. Dwij, A. Khandelwal, M. K. Chattopadhyay, V. Sathe, R. Mittal, P. U. Sastry, S. N. Achary, A. K. Tyagi, P. D. Babu, M. D. Le, D. Bansal, Evidence of strong orbital-selective spin-orbital-phonon coupling in $CrVO_4$. *Phys. Rev. Lett.* **132**, 026701 (2024).

17. M. Fiebig, T. Lottermoser, D. Meier, M. Trassin, The evolution of multiferroics. *Nat. Rev. Mater.* **1**, 16046 (2016).

18. S. J. Whiteley, G. Wolfowicz, C. P. Anderson, A. Bourassa, H. Ma, M. Ye, G. Koolstra, K. J. Satzinger, M. V. Holt, F. J. Heremans, A. N. Cleland, D. I. Schuster, G. Galli, D. D. Awschalom, Spin–phonon interactions in silicon carbide addressed by Gaussian acoustics. *Nat. Phys.* **15**, 490–495 (2019).

19. P. Caravan, Strategies for increasing the sensitivity of gadolinium based MRI contrast agents. *Chem. Soc. Rev.* **35**, 512–523 (2006).

20. A. Lunghi, S. Sanvito, How do phonons relax molecular spins? *Sci. Adv.* **5**, eaax7163 (2019).

21. I. Stockem, A. Bergman, A. Glensk, T. Hickel, F. Körmann, B. Grabowski, J. Neugebauer, B. Alling, Anomalous phonon lifetime shortening in paramagnetic CrN caused by spin-lattice coupling: A combined spin and Ab Initio molecular dynamics study. *Phys. Rev. Lett.* **121**, 125902 (2018).

22. L. Casillas-Trujillo, R. Armiento, B. Alling, Identification of materials with strong magnetostructural coupling using computational high-throughput screening. *Phys. Rev. Mater.* **5**, 034417 (2021).

23. C. Constantin, M. B. Haider, D. Ingram, A. R. Smith, Metal/semiconductor phase transition in chromium nitride(001) grown by rf-plasma-assisted molecular-beam epitaxy. *Appl. Phys. Lett.* **85**, 6371–6373 (2004).

24. B. Biswas, S. Chakraborty, A. Joseph, S. Acharya, A. I. K. Pillai, C. Narayana, V. Bhatia, M. Garbrecht, B. Saha, Secondary phase limited metal-insulator phase transition in chromium nitride thin films. *Acta Mater.* **227**, 117737 (2022).

25. P. A. Bhobe, A. Chainani, M. Taguchi, T. Takeuchi, R. Eguchi, M. Matsunami, K. Ishizaka, Y. Takata, M. Oura, Y. Senba, H. Ohashi, Y. Nishino, M. Yabashi, K. Tamasaku, T. Ishikawa, K. Takenaka, H. Takagi, S. Shin, Evidence for a correlated insulator to antiferromagnetic metal transition in CrN. *Phys. Rev. Lett.* **104**, 236404 (2010).

26. A. Filippetti, N. A. Hill, Magnetic stress as a driving force of structural distortions: The case of CrN. *Phys. Rev. Lett.* **85**, 5166–5169 (2000).

27. B. Biswas, S. Rudra, R. S. Rawat, N. Pandey, S. Acharya, A. Joseph, A. I. K. Pillai, M. Bansal, M. de h-Óra, D. P. Panda, A. B. Dey, F. Bertram, C. Narayana, J. MacManus-Driscoll, T. Maity, M. Garbrecht, B. Saha, Magnetic stress-driven metal-insulator transition in strongly correlated antiferromagnetic CrN. *Phys. Rev. Lett.* **131**, 126302 (2023).

28. B. Biswas, S. Chakraborty, O. Chowdhury, D. Rao, A. I. K. Pillai, V. Bhatia, M. Garbrecht, J. P. Feser, B. Saha, In-plane Cr_2N-CrN metal-semiconductor heterostructure with improved thermoelectric properties. *Phys. Rev. Mater.* **5**, 114605 (2021).

29. C. X. Quintela, B. Rodríguez-González, F. Rivadulla, Thermoelectric properties of heavy-element doped CrN. *Appl. Phys. Lett.* **104**, 022103 (2014).

30. O. Jankovský, D. Sedmidubský, Š. Huber, P. Šimek, Z. Sofer, Synthesis, magnetic and transport properties of oxygen-free CrN ceramics. *J. Eur. Ceram. Soc.* **34**, 4131–4136 (2014).

31. P. Tomeš, D. Logvinovich, J. Hejtmánek, M. H. Aguirre, A. Weidenkaff, Magnetic influence on thermoelectric properties of $CrO_{0.1}N_{0.9}$. *Acta Mater.* **59**, 1134–1140 (2011).

32. T. M. Tritt, *Thermal Conductivity: Theory, Properties, and Applications* (Springer Science & Business Media, 2005).

33. D. T. Morelli, G. A. Slack, "High lattice thermal conductivity solids," in *High Thermal Conductivity Materials* (Springer, 2006), pp. 37–68.

34. P. A. Sharma, J. S. Ahn, N. Hur, S. Park, S. B. Kim, S. Lee, J.-G. Park, S. Guha, S.-W. Cheong, Thermal conductivity of geometrically frustrated, ferroelectric $YMnO_3$: Extraordinary spin-phonon interactions. *Phys. Rev. Lett.* **93**, 177202 (2004).

35. D. G. Tomuta, S. Ramakrishnan, G. J. Nieuwenhuys, J. A. Mydosh, The magnetic susceptibility, specific heat and dielectric constant of hexagonal $YMnO_3$, $LuMnO_3$ and $ScMnO_3$. *J. Phys. Condens. Matter* **13**, 4543 (2001).

36. S. Kielar, C. Li, H. Huang, R. Hu, C. Slebodnick, A. Alatas, Z. Tian, Anomalous lattice thermal conductivity increase with temperature in cubic GeTe correlated with strengthening of second-nearest neighbor bonds. *Nat. Commun.* **15**, 6981 (2024).

37. B. Skubic, J. Hellsvik, L. Nordström, O. Eriksson, A method for atomistic spin dynamics simulations: Implementation and examples. *J. Phys. Condens. Matter* **20**, 315203 (2008).

38. V. P. Antropov, M. I. Katsnelson, B. N. Harmon, M. van Schilfgaarde, D. Kusnezov, Spin dynamics in magnets: Equation of motion and finite temperature effects. *Phys. Rev. B* **54**, 1019–1035 (1996).

39. L. M. Corliss, N. Elliott, J. M. Hastings, Antiferromagnetic structure of CrN. *Phys. Rev.* **117**, 929–935 (1960).

40. A. Q. R. Baron, Y. Tanaka, S. Goto, K. Takeshita, T. Matsushita, T. Ishikawa, An x-ray scattering beamline for studying dynamics. *J. Phys. Chem. Solid* **61**, 461–465 (2000).

41. H. Uchiyama, Y. Oshima, R. Patterson, S. Iwamoto, J. Shiomi, K. Shimamura, Phonon lifetime observation in epitaxial ScN film with inelastic x-ray scattering spectroscopy. *Phys. Rev. Lett.* **120**, 235901 (2018).

42. Z. Gui, C. Gu, H. Cheng, J. Zhu, X. Yu, E. Guo, L. Wu, J. Mei, J. Sheng, J. Zhang, J. Wang, Y. Zhao, L. Bellaiche, L. Huang, S. Wang, Improper multiferroiclike transition in a metal. *Phys. Rev. B* **105**, L180101 (2022).

43. C. Kittel, P. McEuen, *Introduction to Solid State Physics* (John Wiley & Sons, 2018).

44. L. Zhou, F. Körmann, D. Holec, M. Bartosik, B. Grabowski, J. Neugebauer, P. H. Mayrhofer, Structural stability and thermodynamics of CrN magnetic phases from ab initio calculations and experiment. *Phys. Rev. B* **90**, 184102 (2014).

45. C. X. Quintela, F. Rivadulla, J. Rivas, Thermoelectric properties of stoichiometric and hole-doped CrN. *Appl. Phys. Lett.* **94**, 152103 (2009).

46. K. Ahmeda, B. Ubochi, B. Benbakhti, S. J. Duffy, A. Soltani, W. D. Zhang, K. Kalna, Role of self-heating and polarization in AlGaN/GaN-based heterostructures. *IEEE Access* **5**, 20946–20952 (2017).

47. H. Fukui, T. Katsura, T. Kuribayashi, T. Matsuzaki, A. Yoneda, E. Ito, Y. Kudoh, S. Tsutsui, A. Q. R. Baron, Precise determination of elastic constants by high-resolution inelastic x-ray scattering. *J. Synchrotron Radiat.* **15**, 618–623 (2008).

48. G. Kresse, Ab initio molecular dynamics for liquid metals. *J. Non Cryst. Solids* **192-193**, 222–229 (1995).

49. G. Kresse, J. Hafner, Ab initio molecular-dynamics simulation of the liquid-metal–amorphous-semiconductor transition in germanium. *Phys. Rev. B* **49**, 14251–14269 (1994).

50. G. Kresse, J. Furthmüller, Efficient iterative schemes for ab initio total-energy calculations using a plane-wave basis set. *Phys. Rev. B* **54**, 11169–11186 (1996).

51. A. Lindmaa, R. Lizárraga, E. Holmström, I. A. Abrikosov, B. Alling, Exchange interactions in paramagnetic amorphous and disordered crystalline CrN-based systems. *Phys. Rev.* **88**, 054414 (2013).

52. G. Kresse, D. Joubert, From ultrasoft pseudopotentials to the projector augmented-wave method. *Phys. Rev. B* **59**, 1758–1775 (1999).

53. P. E. Blöchl, Projector augmented-wave method. *Phys. Rev. B* **50**, 17953–17979 (1994).

54. S. L. Dudarev, G. A. Botton, S. Y. Savrasov, C. J. Humphreys, A. P. Sutton, Electron-energy-loss spectra and the structural stability of nickel oxide: An LSDA+U study. *Phys. Rev. B* **57**, 1505–1509 (1998).

55. P.-W. Ma, S. L. Dudarev, Constrained density functional for noncollinear magnetism. *Phys. Rev. B* **91**, 054420 (2015).

56. S. Wang, X. Yu, J. Zhang, M. Chen, J. Zhu, L. Wang, D. He, Z. Lin, R. Zhang, K. Leinenweber, Y. Zhao, Experimental invalidation of phase-transition-induced elastic softening in CrN. *Phys. Rev. B* **86**, 64111 (2012).

57. H. J. Monkhorst, J. D. Pack, Special points for Brillouin-zone integrations. *Phys. Rev. B* **13**, 5188–5192 (1976).

58. A. Glensk, B. Grabowski, T. Hickel, J. Neugebauer, J. Neuhaus, K. Hradil, W. Petry, M. Leitner, Phonon lifetimes throughout the brillouin zone at elevated temperatures from experiment and *Ab Initio*. *Phys. Rev. Lett.* **123**, 235501 (2019).

59. S.-H. Wei, L. G. Ferreira, J. E. Bernard, A. Zunger, Electronic properties of random alloys: Special quasirandom structures. *Phys. Rev. B* **42**, 9622–9649 (1990).

60. B. Alling, T. Marten, I. A. Abrikosov, Effect of magnetic disorder and strong electron correlations on the thermodynamics of CrN. *Phys. Rev. B* **82**, 184430 (2010).

61. A. Togo, L. Chaput, T. Tadano, I. Tanaka, Implementation strategies in phonopy and phono3py. *J. Phys. Condens. Matter* **35**, 353001 (2023).

62. C. X. Quintela, J. P. Podkaminer, M. N. Luckyanova, T. R. Paudel, E. L. Thies, D. A. Hillsberry, D. A. Tenne, E. Y. Tsymbal, G. Chen, C. B. Eom, F. Rivadulla, Epitaxial CrN thin films with high thermoelectric figure of merit. *Adv. Mater.* **27**, 3032–3037 (2015).

63. A.-M. Zieschang, J. D. Bocarsly, M. Dürrschnabel, H.-J. Kleebe, R. Seshadri, B. Albert, Low-temperature synthesis and magnetostructural transition in antiferromagnetic, refractory nanoparticles: Chromium nitride, CrN. *Chem. Mater.* **30**, 1610–1616 (2018).

64. R. Wang, Z. Liu, Xianglian, Y. Sun, W. Xia, Analysis of atomic thermal vibration of CrN based on rietveld refinement method. *Phys. Status Solidi B* **261**, 2300195 (2024).

65. D. Gall, C.-S. Shin, R. T. Haasch, I. Petrov, J. E. Greene, Band gap in epitaxial NaCl-structure CrN(001) layers. *J. Appl. Phys.* **91**, 5882–5886 (2002).

66. S. Kalal, S. Nayak, S. Sahoo, R. Joshi, R. J. Choudhary, R. Rawat, M. Gupta, Electronic correlations in epitaxial CrN thin film. *Sci. Rep.* **13**, 15994 (2023).

67. M. A. Gharavi, S. Kerdsongpanya, S. Schmidt, F. Eriksson, N. V. Nong, J. Lu, B. Balke, D. Fournier, L. Belliard, A. le Febvrier, C. Pallier, P. Eklund, Microstructure and thermoelectric properties of CrN and CrN/Cr_2N thin films. *J. Phys. D Appl. Phys.* **51**, 355302 (2018).

68. A. le Febvrier, D. Gambino, F. Giovannelli, B. Bakhit, S. Hurand, G. Abadias, B. Alling, P. Eklund, *p*-type behavior of CrN thin films via control of point defects. *Phys. Rev. B* **105**, 104108 (2022).
